# The C0-C1f Region of Cardiac Myosin Binding Protein-C Induces Pro-Inflammatory Responses in Fibroblasts via TLR4 Signaling

**DOI:** 10.3390/cells10061326

**Published:** 2021-05-26

**Authors:** Athiththan Yogeswaran, Christian Troidl, James W. McNamara, Jochen Wilhelm, Theresa Truschel, Laila Widmann, Muhammad Aslam, Christian W. Hamm, Sakthivel Sadayappan, Christoph Lipps

**Affiliations:** 1 Medical Clinics I—Cardiology and Angiology, Justus-Liebig-University, 35392 Giessen, Germany; Athiththan.Yogeswaran@innere.med.uni-giessen.de (A.Y.); christian.troidl@innere.med.uni-giessen.de (C.T.); laila.widmann@innere.med.uni-giessen.de (L.W.); muhammad.aslam@physiologie.med.uni-giessen.de (M.A.); christian.hamm@innere.med.uni-giessen.de (C.W.H.); 2 German Center for Cardiovascular Research e.v. (DZHK), Partnersite RhineMain, 61231 Bad Nauheim, Germany; 3 Kerckhoff Heart and Thorax Center, Department of Cardiology, 61231 Bad Nauheim, Germany; 4 Department of Internal Medicine, Heart, Lung and Vascular Institute, Division of Cardiovascular Health and Sciences, University of Cincinnati College of Medicine, Cincinnati, OH 45267, USA; james.mcnamara@mcri.edu.au (J.W.M.); SADAYASL@ucmail.uc.edu (S.S.); 5 Murdoch Children’s Research Institute and Melbourne Centre for Cardiovascular Genomics and Regenerative Medicine, The Royal Children’s Hospital, Parkville, VIC 3052, Australia; 6 Department of Anatomy and Physiology, School of Biomedical Sciences, The University of Melbourne, Parkville, VIC 3052, Australia; 7 Department of Internal Medicine, Justus-Liebig-University Giessen, 35390 Giessen, Germany; jochen.wilhelm@innere.med.uni-giessen.de; 8 Universities of Giessen and Marburg Lung Center (UGMLC), Institute for Lung Health (ILH), Member of the German Center for Lung Research (DZL), 35392 Giessen, Germany; 9 Inscreenex GmbH, 38124 Brunswick, Germany; theresa.truschel@inscreenex.com

**Keywords:** fibroblasts, inflammation, MYBPC3, C0-C1f, cMyBP-C, miRNA-146

## Abstract

Myocardial injury is associated with inflammation and fibrosis. Cardiac myosin-binding protein-C (cMyBP-C) is cleaved by µ-calpain upon myocardial injury, releasing C0-C1f, an *N*-terminal peptide of cMyBP-C. Previously, we reported that the presence of C0-C1f is pathogenic within cardiac tissue and is able to activate macrophages. Fibroblasts also play a crucial role in cardiac remodeling arising from ischemic events, as they contribute to both inflammation and scar formation. To understand whether C0-C1f directly modulates fibroblast phenotype, we analyzed the impact of C0-C1f on a human fibroblast cell line in vitro by performing mRNA microarray screening, immunofluorescence staining, and quantitative real-time PCR. The underlying signaling pathways were investigated by KEGG analysis and determined more precisely by targeted inhibition of the potential signaling cascades in vitro. C0-C1f induced pro-inflammatory responses that might delay TGFβ-mediated myofibroblast conversion. TGFβ also counteracted C0-C1f-mediated fibroblast activation. Inhibition of TLR4 or NFκB as well as the delivery of miR-146 significantly reduced C0-C1f-mediated effects. In conclusion, C0-C1f induces inflammatory responses in human fibroblasts that are mediated via TRL4 signaling, which is decreased in the presence of TGFβ. Specific targeting of TLR4 signaling could be an innovative strategy to modulate C0-C1f-mediated inflammation.

## 1. Introduction

Heart failure is one of the most common causes of mortality upon hospitalization after myocardial infarction (MI) [1]. Following MI, overwhelming inflammation and formation of fibrotic scars result in pathological ventricular remodeling which is contributor towards both diastolic and systolic heart failure [2,3]. Different cell types are involved in the regulation of described post-MI remodeling [3,4]. In this context, fibroblasts are particularly important due to their abundance in myocardial tissue and their ability to produce and react to inflammatory cytokines as well as vasoactive and pro-fibrotic peptides [5]. Once activated, fibroblasts can produce cytokines such as tumor necrosis factor α (TNFα), matrix metalloproteases (MMP) such as MMP-9 and chemokines such as CXCL-1 and CCL-2 [6]. Thus, fibroblasts preserve and even enhance the inflammatory microenvironment [3,6]. Moreover, increasing concentrations of transforming growth factor β (TGFβ) released by inflammatory cells after myocardial ischemia result in transdifferentiation of fibroblasts to myofibroblasts, which increase collagen production and release [3,6,7]. Furthermore, transdifferentiation is accompanied by increased levels of intracellular α-smooth muscle actin (αSMA) levels encoded by actin α2 (ACTA2) [8,9]. Thereby, fibroblasts regulate scar formation and myocardial stiffening, which are prognostic determinants in patients with heart failure [10,11]. Interfering with the underlying signaling pathways could be a promising therapeutic approach.

Cardiac myosin binding protein-C (cMyBP-C) has recently been described as a potential biomarker for the detection of MI [12]. C0-C1f, its *N*-terminal region, is the major released peptide from cMyBP-C via µ-calpain-dependent active cleavage shortly after myocardial ischemia due to increased intracellular Ca^2+^ levels [13]. Studies demonstrated the importance of C0-C1f in the regulation of the post-MI processes: both, inflammation and fibrosis seem to be regulated by C0-C1f [14,15,16]. Accordingly, inhibition of the effects of C0-C1f may comprise a way to limit unfavorable scarring in post-MI patients. 

However, neither the direct effect of C0-C1f on fibroblasts nor the specific regulation of the pathways induced have been evaluated to date. Therefore, we investigated the role of C0-C1f as an activator of fibroblasts and tested the applicability of miRNA delivery as a potential therapeutic tool for modulating C0-C1f-mediated effects.

## 2. Materials and Methods

### 2.1. Cell Culture and Stimulation of Human Fibroblasts

Immortalized human fibroblasts (huFib, Inscreenex, Braunschweig, Germany) were cultured at 37 °C with 5% CO_2_ in huFib medium (Inscreenex) [17]. Twenty-four hours before adding the stimulation reagent for a specific duration, cells were maintained under serum-free conditions (Serum-Free huFib Medium, Inscreenex). Individual experiments were performed at different passages (p21–p35) of the cell line under serum-free conditions on collagen coated cell culture dishes. Each experiment (*n*) was performed independently if not otherwise stated.

TLR-4 inhibitor (1 µM) (CLI-095, Invivogen, San Diego, CA, USA) or NFκB inhibitor (10 nM) (Bay11-7085, Cayman Chemical, Ann Arbor, MI, USA) were added to the serum-free medium 18 h prior to and concurrently together with the stimulation reagent, as mentioned below.

Transfection of fibroblasts with miRNA-146 mimics (Qiagen, Hilden, Germany) or antimiRNA-146 (miRCURY LNA miRNA Inhibitors, Qiagen) was conducted using Purefection transfection reagent (System Biosciences, Palo Alto, CA, USA) according to the manufacturer’s instructions. 

### 2.2. Peptide Production

Recombinant N-terminal peptides of cMyBP-C used for stimulation were produced as described earlier [14]. In brief, cDNA encoding for C0-C1f and C0-Linker, respectively, were cloned into the Pet28a(+) expression vector (Novagen, Madison, WI, USA) and transformed into BL21 (DE3) *Escherichia coli*. C0-C1f contains the C0-, linker-, C1 domain and first 17 residues of the M-domain, whereas C0-Linker contains C0- and linker-domain of the N-terminal region of cMyBP-C [14]. All coding sequences were cloned in frame with the N-terminal 6xHis Tag and contained a C-terminal termination codon. After induction with isopropyl-β-D-thiogalactopyranoside (IPTG, Roche, Indianapolis, IN, USA) the cells were harvested, and overexpressed proteins were purified via Ni-NTA agarose chromatography according to the manufacturer’s instructions (Qiagen). Lipopolysaccharides from *Escherichia coli* (LPS, Sigma-Aldrich, Hamburg, Germany) and human TGFβ1 (Peprotech, Hamburg, Germany) were used as positive controls.

### 2.3. Immunofluorescence Staining

huFib cells were grown on collagen-coated chamber slides under serum-free conditions. For analysis, cells were fixed for 10 min with 4% paraformaldehyde (PFA) in phosphate-buffered saline. Cells were stained with a mouse monoclonal αSMA antibody conjugated with Cy3 (clone 1A4, Sigma-Aldrich). Nuclei were stained using DAPI (Roth, Karlsruhe, Germany). All images were obtained using a BZ-9000 microscope (Keyence, Neu-Isenburg, Germany).

### 2.4. RNA Isolation and Quantitative Real-Time Polymerase Chain Reaction (qPCR)

Total RNA extraction was performed using the RNeasy mini-kit (Qiagen). cDNA synthesis was conducted using the iScript^TM^ cDNA synthesis kit (BioRad Laboratories, Munich, Germany) according to manufacturer’s instructions. Quantitative real-time PCR (qPCR) was performed using SsoFast^TM^EvaGreen^®^ Mastermix (BioRad Laboratories, Munich, Germany) and the CFX96 real-time PCR system (BioRad Laboratories, Munich, Germany). Glyceraldehyde-3-phosphate dehydrogenase (GAPDH) was used as a housekeeping gene for normalization. Data are presented as log_2_ fold changes relative to controls using the ∆∆Ct method. Primer sequences are shown in Table 1.

### 2.5. Gene Expression Profiling

Purified total RNA was amplified and Cy3-labeled using the LIRAK kit (Agilent Technologies, Santa Clara, CA, USA) following the kit instructions. Per reaction, 200 ng of total RNA was used. The Cy3-labeled aRNA was hybridized overnight to 8 × 60 K 60-mer oligonucleotide spotted microarray slides (Agilent Technologies, design ID 072363). Hybridization and subsequent washing and drying of the slides was performed following the Agilent hybridization protocol.

The dried slides were scanned at 2 µm/pixel resolution using the InnoScan is 900 (Innopsys, Carbonne, France). Image analysis was performed with Mapix 6.5.0 software, and calculated values for all spots were saved as GenePix results files. Stored data were evaluated using the R software [18] and the limma package [19] from BioConductor [20]. Mean spot signals were corrected for background with an offset of 1 using the NormExp procedure on the negative control spots. The logarithms of the background-corrected values were quantile-normalized [19,21]. The normalized values were then averaged for replicate spots per array. From different probes addressing the same NCBI gene ID, the probe showing the maximum average signal intensity over the samples was used in subsequent analyses. Genes were ranked for differential expression levels using moderated t-statistics [19]. Pathway analyses were conducted using gene set tests on the ranks of the t-values [19]. For the KEGG analysis, gene set tests [22] were performed by mean-rank gene-set enrichment using absolute t-statistics [23] on gene sets defined based on the KEGG database [24].

### 2.6. Statistical Analysis

All statistical analyses were performed using R (The R Foundation, Vienna, Austria) or GraphPad software (GraphPad, La Jolla, CA, USA). Mean data are presented as mean ± standard deviation (SD). Unless otherwise stated, unpaired t-tests with pooled variance were utilized to compare control and experimental data. *p* < 0.05 was considered statistically significant.

## 3. Results

### 3.1. Conversion to Myofibroblasts and Collagen Production Is Confined to TGFβ Signaling

**Figure 1:**
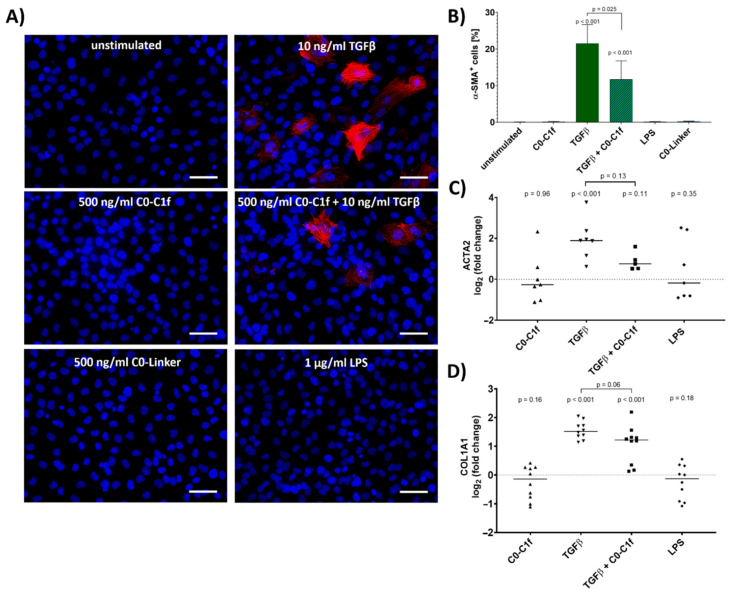
Conversion to myofibroblasts. huFib cells were treated with 10 ng/mL TGFβ, 500 ng/mL C0-C1f, a combination of C0-C1f and TGFβ, 500 ng/mL C0-Linker, or 1 µg/mL LPS. (**A**) Nuclei (blue) and α-SMA (red) were stained after 48 h of treatment under serum-free conditions. Scale bar = 50 µm (**B**) Quantification of α-SMA-positive cells, showing mean and SD (unstimulated *n* = 6, C0-C1f *n* = 3, TGFβ *n* = 5, TGFβ + C0-C1f *n* = 4, LPS *n* = 3, C0-Linker *n* = 3; *n* represents technical replicates). (**C**,**D**) Quantification of mRNA expression levels of ACTA2 and COL1A1 after 24 h treatment by qPCR. Data are displayed as individual data points and means; *p*-values above each condition refer to differences between means (horizontal lines) and 0 fold change (unstimulated controls) unless otherwise indicated. (ACTA2 *n* = 7, TGFβ + C0-C1f *n* = 5; COL1A1 *n* = 10; *n* represents biological replicates).

To evaluate the impact of C0-C1f on the fibrotic response, huFib cells were treated with C0-C1f and other agents for 48 h. C0-Linker did not induce inflammatory responses in macrophages [14] and was used as negative control in the following experiments. Whereas incubation with TGFβ led to increased numbers of αSMA-positive cells, C0-C1f, C0-Linker, or LPS treatment did not increase αSMA abundance within 48 h under serum-free conditions (Figure 1A,B). Of note, costimulation of TGFβ and C0-C1f showed lower numbers of αSMA-positive cells and reduced ACTA2 mRNA expression compared with huFib cells stimulated with TGFβ alone (Figure 1B,C). A similar, albeit less pronounced pattern was observed when comparing collagen (COL1A1) mRNA levels of huFib cells stimulated with TGFβ or TGFβ and C0-C1f (Figure 1D).

### 3.2. C0-C1f Modulates Gene Expression in Fibroblasts Consistent with an Inflammatory Phenotype

**Figure 2:**
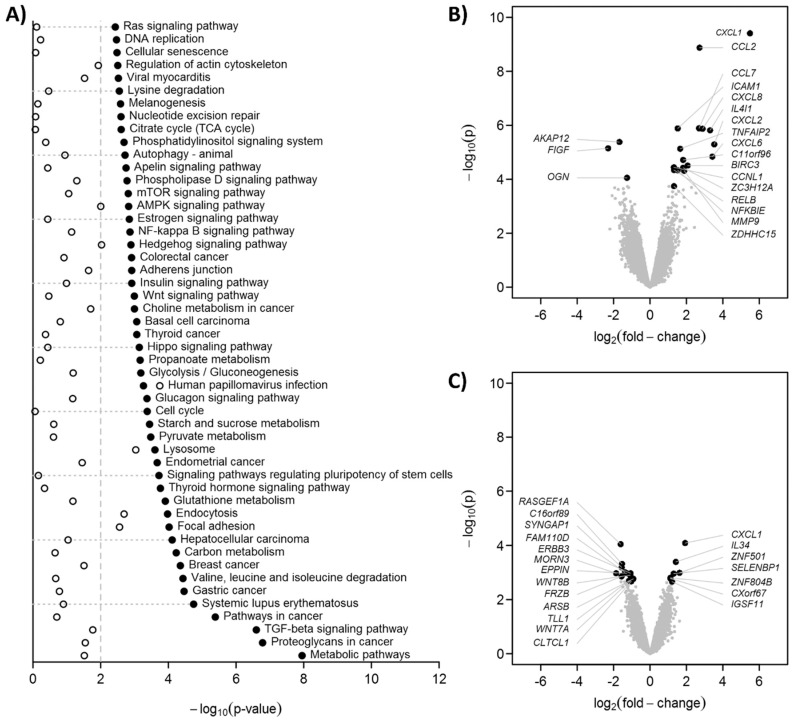
Gene expression profiling. huFib were stimulated with 500 ng/mL C0-C1f, 500 ng/mL C0-Linker for 24h. As controls (Ctrl) unstimulated huFib were harvested. Total mRNA was isolated and microarray analyses performed. (**A**) Statistical significances of the KEGG pathway analyses as −log_10_(*p*). The 50 pathways with lowest *p*-values in C0-C1f vs. control were selected. The vertical dashed line indicates *p* = 0.01. Black dots: C0-C1f vs. control, open circles: C0-linker vs. control. (**B**,**C**) Volcano plots for C0-C1f vs. control and C0-linker vs. control, respectively. The 20 genes with the lowest *p*-values are annotated.

Treatment for 24 h with C0-C1f led to changes in the transcriptome of cultured huFib cells (Figure 2). KEGG analysis revealed that the TGFβ signaling pathway and metabolic pathways, among others, were significantly modulated by C0-C1f (Figure 2A). C0-Linker had only a limited effect on these pathways (Figure 2A). C0-C1f induced primarily inflammation-associated proteins and modulated the NFκB signaling pathway (Figure 2A,B). Expression of chemokines including CXCL-1, CCL-2, CCL-7, CXCL-8, and CXCL-2, as well as ICAM-1 and MMP-9, were significantly upregulated upon C0-C1f treatment (Figure 2B). In contrast, C0-Linker had a low impact on the induction of these factors at the transcriptome level in fibroblasts except for CXCL-1, which was significantly upregulated (Figure 2C). KEGG analysis suggested that the NFκB signaling pathway is involved in the regulation of these observed changes (Figure 2A). 

**Figure 3:**
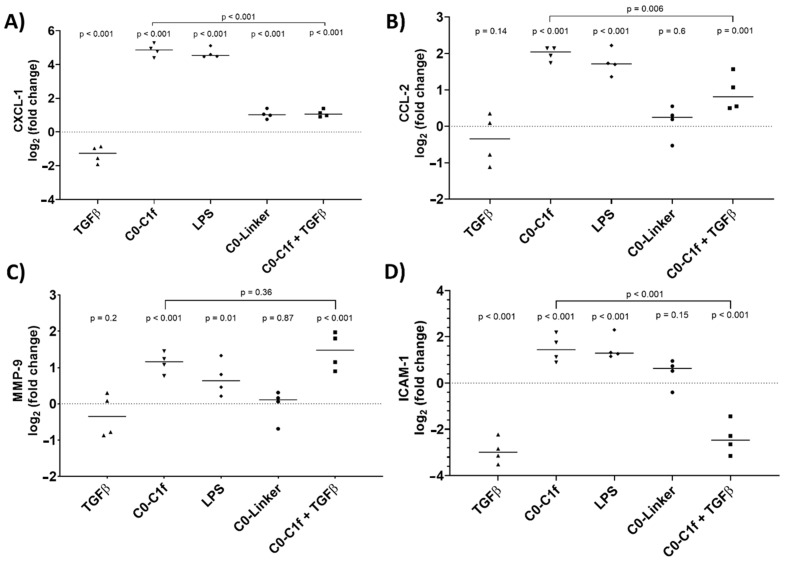
Activation of fibroblasts. huFib cells were treated with 1 µg/mL LPS, 500 ng/mL C0-Linker, or 500 ng/mL C0-C1f and/or 10 ng/mL TGFβ. (**A**) CXCL-1 (**B**) CCL-2 (**C**) MMP-9, and (**D**) ICAM-1 mRNA abundance was detected by qPCR after 24 h treatment. Data are displayed as individual data points and means; *p*-values above each condition refer to differences between means (horizontal lines) and 0 fold change (unstimulated controls) unless otherwise indicated. (*n* = 4; *n* represents biological replicates).

qPCR analysis was performed to validate the major findings of the microarray study. Thus, huFib cells were stimulated for 24 h with TGFβ and/or C0-C1f. CXCL-1 and CCL-2 mRNA was significantly upregulated upon treatment with C0-C1f (Figure 3A,B). MMP-9 and ICAM-1 mRNA was also induced following C0-C1f stimulation (Figure 3C,D). 

In-depth analysis revealed that TGFβ counteracts C0-C1f-mediated signaling: co-stimulation with TGFβ and C0-C1f showed less intense induction of inflammatory chemokines than C0-C1f alone, signifying that TGFβ cotreatment significantly reduced the C0-C1f-mediated induction of these genes (Figure 3A–D). LPS induced the inflammatory markers as well, whereas C0-Linker did not significantly regulate CCL-2, MMP-9, or ICAM-1 expression (Figure 3). 

### 3.3. Time-Dependent Response of Fibroblasts After Stimulation with C0-C1f

**Figure 4:**
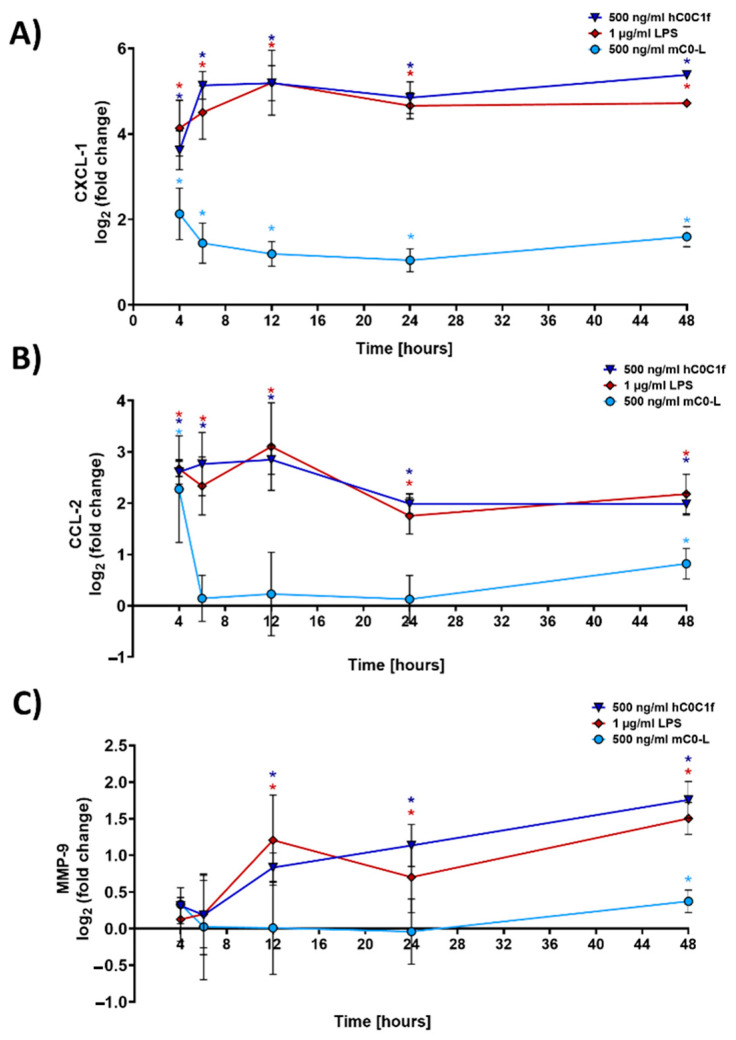
Time-dependent response of fibroblasts. huFib cells were treated with 500 ng/mL C0-C1f, 500 ng/mL C0-Linker or 1 µg/mL LPS for different time periods, and (**A**) CXCL-1, (**B**) CCL-2, and (**C**) MMP-9 mRNA abundance was quantified by qPCR. Data are displayed as mean ± SD (*n* = 4 biologically independent experiments). * above each condition refer to differences between means (data points) and 0 fold change (unstimulated controls). * *p* < 0.05 was considered statistically significant.

The time course of transcriptional changes was assessed by qPCR after 4, 6, 8, 12, 24 and 48 h of stimulation with C0-C1f, C0-Linker, or LPS. Both CXCL-1 and CCL-2 showed the greatest mRNA induction after a 6-h treatment with C0-C1f (Figure 4A,B). CXCL-1 mRNA abundance remained constant during treatment between 8 and 48 h, but CCL-2 mRNA abundance continuously declined after its initial peak (Figure 4A,B). LPS also induced CXCL-1 and CCL-2 mRNA at all observed time points (Figure 4A,B). MMP-9 mRNA abundance increased with time when stimulated with C0-C1f (Figure 4C). A similar time-dependent induction of MMP-9 mRNA expression was observed with LPS stimulation (Figure 4C), whereas C0-Linker had no effect on MMP9 or CCL2 expression (Figure 4B,C).

### 3.4. C0-C1f Signaling Depends on Toll-Like Receptor 4 Activation

C0-C1f caused predominantly pro-inflammatory responses, which is in line with the results of KEGG analysis showing that NFκB signaling is significantly influenced by C0-C1f (Figure 2A). Therefore, we investigated intracellular and extracellular signaling pathways by specifically inhibiting different toll-like receptors (TLR), receptor for advanced glycation end products (RAGE), and NFκB.

**Figure 5:**
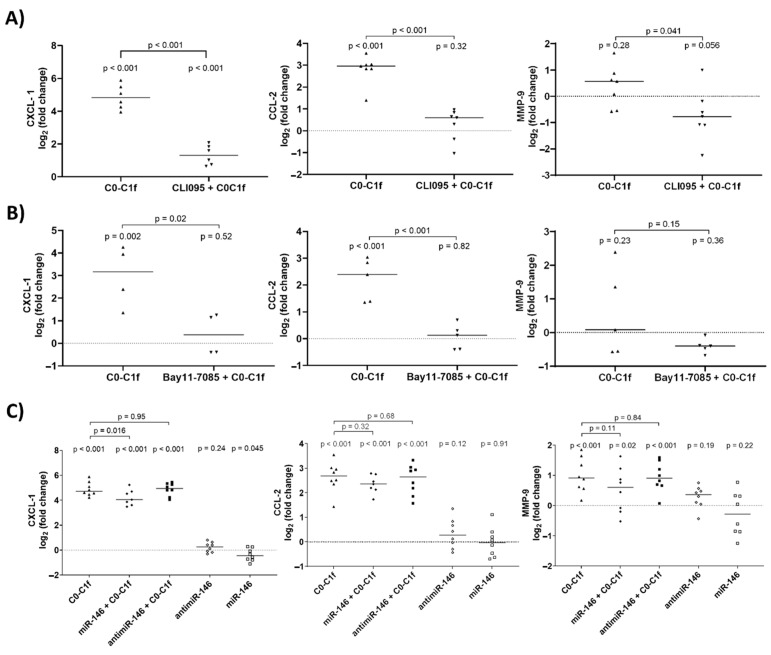
Effects of TLR4 and NFκB inhibition on C0C1f signaling**.** (**A**) huFib cells were pretreated for 18 h with CLI-095 (TLR4 inhibitor) and then stimulated with 500 ng/mL C0-C1f for 6 h. mRNA levels of CXCL-1, CCL-2, and MMP-9 were determined by qPCR and compared using an unpaired t-test. (**B**) huFib cells were pretreated for 18 h with Bay11-7085 (NFκB inhibitor) and stimulated with C0-C1f for 6 h. mRNA levels of CXCL-1, CCL-2, and MMP-9 were determined by qPCR. (**C**) huFib cells were transiently transfected with 25 pmol miR-146 or antimiR-146 oligos for 24 h prior to C0-C1f treatment for 6 h. CXCL-1, CCL-2, and MMP-9 mRNA were detected by qPCR. In all panels *p*-values above each condition refer to differences between means (horizontal lines) and 0 fold change (unstimulated controls) unless otherwise indicated. (**A**) CXCL1 *n* = 6; CCL2 and MMP9 *n* = 7; (**B**) CXCL-1 *n* = 4, CCL2 and MMP9 *n* = 5; (**C**) *n* = 8; n represents biological replicates.

For this purpose, huFib cells were treated with inhibitors for 18 h prior to stimulation. Inhibition of either TLR2 or RAGE did not significantly reduce CXCL-1, CCL-2, or MMP-9 mRNA abundance after C0-C1f treatment (Supplementary Figure S1). However, inhibition of TLR4 or NFκB significantly reduced C0-C1f-mediated CXCL-1 and CCL-2 mRNA induction (Figure 5A,B). Moreover, inhibition of TLR4 or NFκB led to a significant decrease in MMP-9 mRNA abundance when stimulated with C0-C1f (Figure 5A,B). We also tested whether the TLR4-mediated signaling could be disrupted by the delivery of miRNA-146, a well-known inhibitor of TLR4 signaling. For this purpose, huFib cells were transfected with miRNA-146 for 18 h prior to the stimulation with C0-C1f. This led to significantly lower C0-C1f-stimulated CXCL-1 mRNA abundance (Figure 5C), whereas the C0-C1f-stimulated CCL-2 and MMP-9 mRNA abundance was only slightly reduced (Figure 5C). The transfection of huFib cells with antimiR-146 for 18h prior to the stimulation with C0-C1f did not affect the C0-C1f-mediated change in mRNA abundance. Transfection of huFib cells with antimiR-146 or miR-146 alone did not change CXCL-1, CCL-2 or MMP9 mRNA abundance (Figure 5C).

## 4. Discussion

This is the first report showing that a human fibroblast cell line is directly activated by C0-C1f, the *N*-terminal region of cMyBP-C. We demonstrate that C0-C1f induces pro-inflammatory responses in huFib cells but does not promote transdifferentiation of these cells to myofibroblasts within the first 48 h. In fact, C0-C1f reduces pro-fibrotic responses via modulating the TGFβ signaling pathway, as reflected by reduced expression of myofibroblast markers upon costimulation of TGFβ and C0-C1f compared with TGFβ alone (Figure 1). Interestingly, TGFβ also reduced the C0-C1f-mediated pro-inflammatory effects (Figure 3). Our results indicate that the effects of C0-C1f observed in human fibroblasts are dependent on TLR4 and NFκB signaling (Figure 5). 

Ischemia-induced myocardial inflammation and fibrosis are tightly regulated by different signaling mechanisms [3,25]. Identification of mediators of these regulatory mechanisms is of utmost importance to developing novel strategies to increase post-MI survival rates.

C0-C1f has been demonstrated to be released early after myocardial ischemia for a short time period [26]. During this early phase of cardiac remodeling, pro-inflammatory and fibrosis-suppressing processes may be crucial to avoid pathologic scar formation and impaired cardiac function [3]. In line with this, lineage-tracing experiments did not exhibit transdifferentiation of fibroblasts to myofibroblasts until days 4–7 after myocardial infarction [27].

C0-C1f has previously been described to induce both inflammation and fibrosis [14,15,28]. In transgenic mice, cardiomyocyte-specific overexpression of C0-C1f over a timeframe of at least four weeks led to cardiac fibrosis [15]. After induced myocardial ischemia, µ-calpain-dependent proteolysis resulted in the transient release of C0-C1f [13,26].

We addressed here the question whether direct stimulation of fibroblasts leads to fibrosis, as the previously described effects [14] may be due exclusively to activation of other cell types such as macrophages. Although C0-C1f did not cause differentiation of fibroblasts towards the myofibroblast phenotype, characterized by αSMA expression, C0-C1f-dependent activation of fibroblasts caused a pro-inflammatory state, which in the myocardium might actively contribute to remodeling. The induction of chemokines such as CXCL-1 and CCL-2, which we observed to increase upon C0-C1f treatment in fibroblasts, is part of the observed macrophage invasion post-MI [29,30]. In addition, an increase in ICAM-1 levels is common after MI and leads to enhanced neutrophil and monocyte adhesion, which causes the accumulation of immune cells in ischemic regions [31]. Furthermore, we showed that C0-C1f treatment of fibroblasts upregulated the expression of MMP-9 mRNA, which is an important factor in extracellular matrix remodeling in the infarcted myocardium and a key step in the infiltration of inflammatory cells as well as scar formation [32,33,34]. Moreover, MMP-9 cleaves chemokines and thereby silences their effects [35]. Our results showed that MMP-9 mRNA levels steadily increase during a 24 h treatment with C0-C1f. Since C0-Linker did not lead to changes in CCL-2, ICAM-1 or MMP-9 mRNA, we conclude that the observed activation of fibroblasts is specific to the C0-C1f peptide. 

Considering the ability of C0-C1f to activate macrophages, our results are consistent with C0-C1f playing an important role in post-MI remodeling [14]. Both the C0-C1f-mediated increase in chemokine and pro-inflammatory cytokine mRNA expression and the direct interaction with TGFβ signaling may inhibit fibroblast differentiation in early phases of MI. In contrast, we found that TGFβ reduces the pro-inflammatory responses in fibroblasts mediated by C0-C1f. Thus, C0-C1f may be one of the corresponding regulatory counterparts to TGFβ-driven fibrosis within the first days after myocardial infarction. Conversely, TGFβ release in advanced stages of cardiac remodeling post-MI could suppress the inflammatory microenvironment established by C0-C1f due to direct inactivation of pro-inflammatory gene regulation in fibroblasts.

We hypothesize that the previously reported fibrosis in transgenic mice overexpressing C0-C1f is most likely caused by indirect effects of C0-C1f on other cell types [14,15,28]; it is well known that increased concentrations of inflammatory cytokines can activate immune cells such as macrophages, which leads to increased levels of TGFβ, a known cause of fibroblast–myofibroblast transdifferentiation [25,36].

Overwhelming cardiac inflammation is known to cause diastolic dysfunction and heart failure [32,37]. Accordingly, understanding the underlying signaling of C0-C1f-induced activation of fibroblasts is crucial. We showed that C0-C1f triggers predominantly pro-inflammatory responses in fibroblasts via NFκB signaling. TLR4, which is a well-known receptor for danger-associated molecular patterns, is activated by extracellular C0-C1f and contributes to the observed effects, which is in line with previously described signaling pathways of C0-C1f in macrophages [14,38]. In contrast to macrophages, TLR2 and RAGE are not required for the induction of CXCL-1 or CCL-2 upon C0-C1f treatment in fibroblasts [14], which is consistent with the data we obtained using inhibitors of these receptors. 

TLR4 pathways play an important role in the post-MI setting [3,38,39,40]. In vivo studies showed that TLR4 activation during MI increased inflammatory cytokines levels and was associated with larger infarct size due to augmented apoptosis, oxidative stress and maladaptation [3]. Notably, short-term TLR4 activation reduced infarct sizes, suggesting short-term TLR4 activation may be cardioprotective [3]. 

Compounds such as CLI-095 or Bay11-7085 are potent inhibitors of the TLR4 signaling cascade and block the activation of TLR4 itself or the activation of NFκB, respectively, and constitute potential candidates for therapeutic development. However, recent progress in developing in vivo delivery systems for miRNAs brings this class of compounds back to the therapeutic forefront [41]. Administration of miRNAs or anti-miRNAs has been successfully tested in clinical phase I studies, specifically antimiR-92a in cardiovascular disease therapy and antimiR-132 in heart failure patients [41,42,43]. Successful inhibition of TLR4/NFκB signaling in fibroblast-like cells has been demonstrated in vitro by administration of miR-146, which also inhibits pro-fibrotic and inflammatory signaling pathways in renal fibrosis in vivo [44,45].

In the current study, treatment of human fibroblasts with miRNA-146 reduced the impact of C0-C1f on fibroblast activation, as demonstrated by a significant reduction in CXCL-1 mRNA abundance. In a post-MI setting, reduced levels of pro-inflammatory cytokines initiate the switch from the inflammatory phase to the fibrotic phase and scar formation [32]. Therefore, inhibition of C0-C1f-mediated fibroblast activation by miRNA-146 may lead to smaller infarct size since less healthy tissue is degraded [46]. This hypothesis is consistent with recently published data indicating that miRNA-146 attenuates cardiac remodeling and dysfunction during MI [46]. Indeed, recent results obtained in vivo showed improved cardiac function due to reduced NFκB activation and release of cytokines [47].

The model chosen for the present investigation is a human fibroblast cell line that is non-cardiac in origin. The availability of human primary cardiac fibroblast cells from “cardiac healthy donors” is restricted and their expansion in vitro is limited. It is challenging to maintain the “naïve” fibroblast phenotype upon isolation and during cultivation and to avoid myofibroblast transdifferentiation. Thus, here we applied an established immortalized human fibroblast cell line, derived from foreskin that shows robust proliferation and stable phenotype over at least 35 passages. For many applications cell lines represent an attractive alternative to primary cells since they can be characterized in depth and demonstrate unlimited proliferation [48]. However, we are aware that the high proliferation capacity is accompanied by a certain loss of physiological relevance. As such, this model is limited in its capacity to test effects on proliferation kinetics or its application in typically used in vitro “scratch” assays.

## 5. Conclusions

Our results are consistent with the hypothesis that C0-C1f is an important regulator of post-MI remodeling. C0-C1f promotes an inflammatory state in fibroblasts and this might delay their differentiation to myofibroblasts. Moreover, we demonstrate that C0-C1f acts primarily via activation of TLR4 and NFκB. Regulation of C0-C1f signaling and thus modulation of inflammation and fibrosis, which are important determinants of mortality in patients with myocardial ischemia, is a promising approach to increasing survival rates after MI. Application of miRNAs such as miRNA-146 is one possible strategy to modulate the contribution of C0-C1f to cardiac remodeling upon myocardial injury.

## Figures and Tables

**Table 1:** List of primers used for qPCR analysis.

Target	Fwd	Rev
GAPDH	CCTCAAGATCATCAGCAATGCCTCCT	GGTCATGAGTCCTTCACGATACCAA
ACTA2	ACCATGAAGATCAAGATCATTGCC	AAACACATAGGTAACGAGTCAGAG
CXCL1	TCACCCCAAGAACATCCAAAG	TGGATTTGTCACTGTTCAGCA
CCL2	GCAGCAAGTGTCCCAAAGAA	CTGGGGAAAGCTAGGGGAAA
ICAM-1	TTGGGCATAGAGACCCCGTT	GCACATTGCTCAGTTCATACACC
COL1A1	QT00037793 (Hs_COL1A1_1_SG, Qiagen)	
MMP9	QT00040040 (Hs_MMP9_1_SG, Qiagen)	

## Data Availability

The data presented in this study are available on request from the corresponding author.

## Supplementary Materials

The following are available online at https://www.mdpi.com/article/10.3390/cells10061326/s1, Figure S1. C0C1f signaling is not dependent on TLR2 or RAGE, Supplementary Data 1: Microarray data.
