# Supplementary figures and images for: The C0-C1f Region of Cardiac Myosin Binding Protein-C Induces Pro-Inflammatory Responses in Fibroblasts via TLR4 Signaling
Source: Cells. 2021 May 26;10(6):1326. doi: 10.3390/cells10061326 (PMC8230336; doi:10.3390/cells10061326)

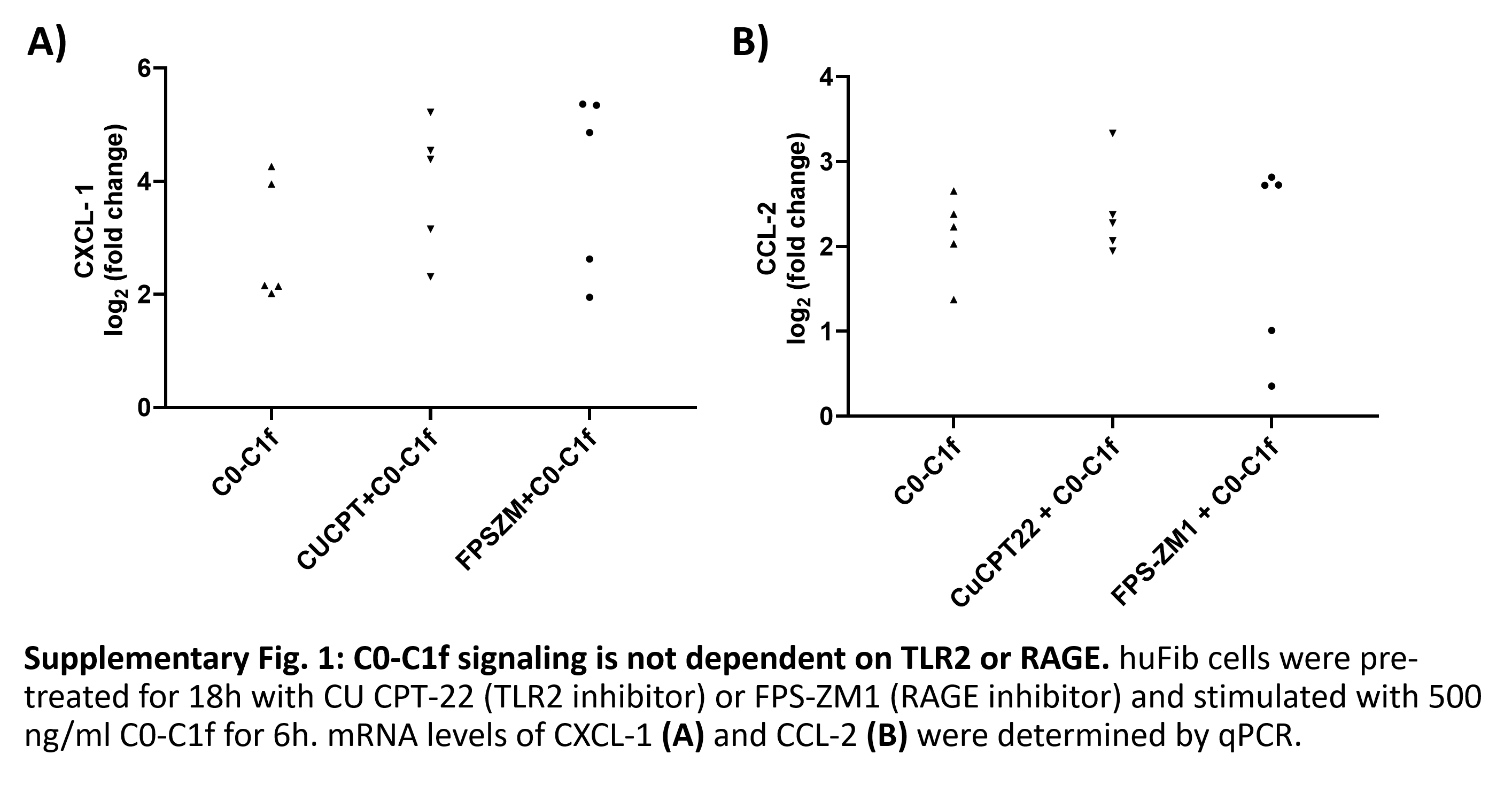

Supplement: Supplementary file 1 [file cells-10-01326-s001.zip › Supplementary Fig.1.tif]
